# Supplementary material for: The causal role of circulating inflammatory markers in osteoporosis: a bidirectional Mendelian randomized study
Source: Front Immunol. 2024 Jul 18;15:1412298. doi: 10.3389/fimmu.2024.1412298 (PMC11291241; doi:10.3389/fimmu.2024.1412298)
Supplement: Supplementary file 1 [file DataSheet_1.zip › supplementary material/CIM-OSTEOPOROSIS /GCST90274797.csv_leave_one_out.pdf]

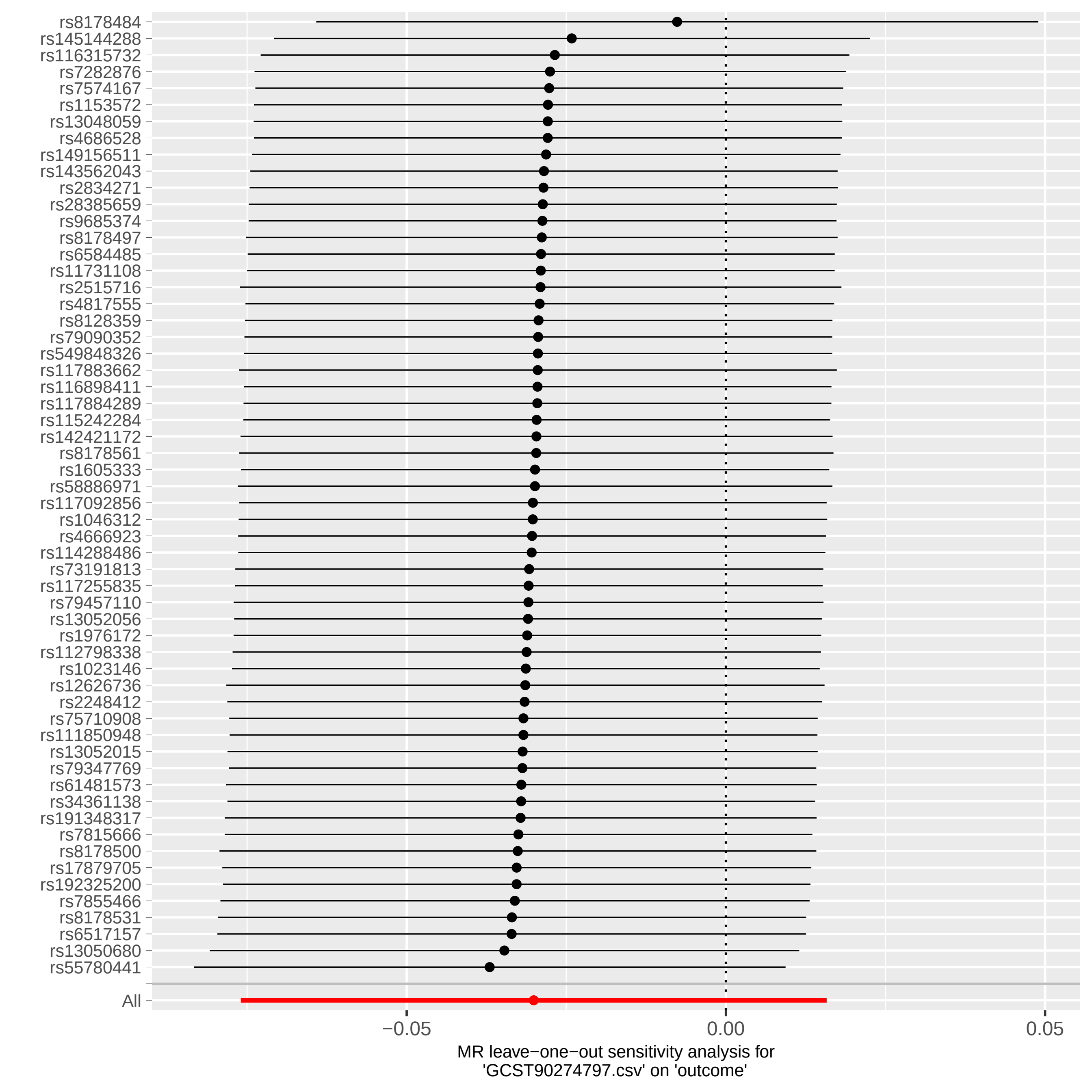

rs8178484
rs145144288
rs116315732
rs7282876
rs7574167
rs1153572
rs13048059
rs4686528
rs149156511
rs143562043
rs2834271
rs28385659
rs9685374
rs8178497
rs6584485
rs11731108
rs2515716
rs4817555
rs8128359
rs79090352
rs549848326
rs117883662
rs116898411
rs117884289
rs115242284
rs142421172
rs8178561
rs1605333
rs58886971
rs117092856
rs1046312
rs4666923
rs114288486
rs73191813
rs117255835
rs79457110
rs13052056
rs1976172
rs112798338
rs1023146
rs12626736
rs2248412
rs75710908
rs111850948
rs13052015
rs79347769
rs61481573
rs34361138
rs191348317
rs7815666
rs8178500
rs17879705
rs192325200
rs7855466
rs8178531
rs6517157
rs13050680
rs55780441
All
−0.05
0.00
0.05
MR leave−one−out sensitivity analysis for
'GCST90274797.csv' on 'outcome'
